# Resilience Factors and Physical Activity Engagement in Adolescents with Chronic Musculoskeletal Pain: A Cross-Sectional Study

**DOI:** 10.3390/jcm14217621

**Published:** 2025-10-27

**Authors:** William R. Black, Haley Hart, Jennifer Christofferson, Mark Connelly, Liesbet Goubert, Dustin P. Wallace, Laura Ellingson-Sayen, Ann M. Davis

**Affiliations:** 1 Department of Pediatrics, The Ohio State University Medical Center, Columbus, OH 43212, USA; 2 Center for Biobehavioral Health, The Abigail Wexner Research Institute, Nationwide Children’s Hospital, 700 Children’s Drive, J West 3rd Floor, Columbus, OH 43215, USA; 3 Department of Clinical Psychology, University of Kanas, Lawrence, KS 66045, USA; 4 Department of Clinical Child Psychology, University of Kanas, Kansas City, MO 66160, USA; 5 Department of Pediatrics, Children’s Mercy Kansas City, Kansas City, MO 64111, USA; 6 Department of Clinical Health Psychology, Gent University, 9000 Ghent, Belgium; 7 Division of Health and Exercise Sciences, Western Oregon University, Monmouth, OR 97361, USA; 8 Department of Pediatrics, University of Kansas Medical Center, Kansas City, MO 66160, USA; 9 Center for Children’s Healthy Lifestyles & Nutrition, Kansas City, MO 64108, USA

**Keywords:** adolescent chronic pain, physical activity, resilience, psychological flexibility, pain acceptance, self-efficacy, quality of life, pain coping, moderate-to-vigorous physical activity

## Abstract

**Background/Objectives**: Chronic musculoskeletal pain (CMSKP) affects up to 40% of adolescents and leads to substantial disability, reduced quality of life, and long-term health risks. Physical activity is central to treatment, but adherence to moderate-to-vigorous physical activity (MVPA) is inconsistent. We evaluated higher-resilience constructs—self-efficacy, pain acceptance, motivational stage, and affect—and hypothesized that higher resilience would be associated with greater objectively measured physical activity, better daily functioning, and higher quality of life in adolescents with CMSKP. **Methods**: Forty-three adolescents (13–18 years) with CMSKP completed measures of physical activity-specific self-efficacy, acceptance (AFQ-Y), motivational stage (PSOCQ-A), and affect (PANAS-C). Participants wore activPAL monitors to assess MVPA, light activity, and sedentary time. Physical function endurance was measured by the six-minute walk test (6MWT) and the Functional Disability Inventory (FDI); quality of life by the Pediatric Quality of Life Inventory (PedsQL). Spearman’s correlations assessed associations among resilience variables, physical activity metrics, 6MWT distance, FDI, and PedsQL. **Results**: MVPA was correlated positively with 6MWT distance (*ρ* = 0.48, *p* = 0.002) and negatively with FDI scores (*ρ* = −0.56, *p* < 0.001). Self-efficacy related to higher MVPA (*ρ* = 0.41, *p* = 0.009), better endurance (*ρ* = 0.36, *p* = 0.017), and lower disability (*ρ* = −0.38, *p* = 0.013). Acceptance was correlated with PedsQL total (*ρ* = 0.45, *p* = 0.004); motivation (specifically maintenance) scores were correlated with higher quality of life (*ρ* = 0.33, *p* = 0.027). Light activity and sedentary time were not significantly linked to functional or psychosocial outcomes. In a step-wise regression, only physical activity self-efficacy for ambulation at school predicted MVPA, *B =* 1.56, *p* = 0.008. **Conclusions**: Resilience constructs—including self-efficacy, acceptance, and readiness to change—were meaningfully associated with MVPA, daily functioning, and quality of life, and may have implications for treatment development.

## 1. Introduction

Chronic musculoskeletal pain (CMSKP)—defined as pain in muscles, ligaments, bones, or joints persisting for three or more months—affects approximately 38–40% of adolescents in the United States [1,2,3] and poses a substantial public health challenge and significant impairment, including increased school absenteeism [4,5] academic difficulties [6], reduced physical functioning, and elevated psychosocial distress [7]. The economic burden associated with pediatric CMSKP exceeds USD 12 billion annually, surpassing healthcare costs related to pediatric obesity and asthma combined [8]. Notably, adolescents experiencing CMSKP are at increased risk for persistent chronic pain into adulthood, which further exacerbates healthcare costs, functional impairments, and overall disability [9,10,11,12].

Multidisciplinary treatments combining psychological interventions and physical activity regimens are the standard of care in pediatric CMSKP [13,14]. Yet, adherence to physical activity regimens is often inadequate, limited by barriers such as avoidance of physical activity due to fear of increased pain (fear–avoidance), low motivation for exercise, and low self-efficacy for engaging in physical activity [15,16,17]. These challenges underscore the importance of identifying modifiable psychosocial factors that can increase sustained physical activity among adolescents with CMSKP. Pain resilience is a promising target to support physical activity adherence and recovery in youth with CMSKP [18,19]. Pain resilience refers to maintaining engagement in valued activities, even when experiencing ongoing pain, and is increasingly understood as a dynamic, context-dependent process that enhances adaptive coping and behavioral persistence in the face of adversity [20,21].

Multiple resilience factors—self-efficacy, pain acceptance, motivational readiness, and positive affect—have been identified as potentially critical to promoting physical activity in chronic pain contexts [19,22]. Self-efficacy for physical activity—confidence in one’s ability to engage in physical activity even when experiencing pain—is among the most robust psychological predictors of physical activity engagement and functional outcomes in chronic pain populations [23,24]. Pain acceptance, reflecting a willingness to pursue meaningful activities without sacrificing those activities or one’s enjoyment of them by attempting to control or eliminate pain, has been associated with reduced functional disability and improved quality of life (QOL) in pediatric intensive interdisciplinary pain treatment (IIPT) [25]. Motivation influences the initiation and persistence of physical activity behaviors, directly linking to sustained treatment adherence [26,27]. Additionally, positive affect acts as a buffer against fear–avoidance patterns, consistent with the broaden-and-build theory [28] that positive emotions broaden flexibility of thoughts and actions and can promote activity persistence despite discomfort [29,30]. Other resilience factors may also be important for understanding pain and outcomes, including optimism [31], grit [32], and a growth mindset [33], but have been studied far less than other resilience factors.

Previous qualitative research conducted by our team has highlighted these resilience processes as critical facilitators of physical activity engagement among adolescents who had previously completed IIPT. Participants consistently reported that enhanced motivation, values-based decision-making, acceptance of pain, and improved self-efficacy were essential to their successful re-engagement in physical activity (In Press) [34]. These qualitative findings underscore the potential clinical utility of resilience-focused approaches for improving physical activity outcomes, yet quantitative studies examining how these constructs influence objectively measured physical activity and physical function in adolescents with CMSKP remain scarce.

**Figure 1:**
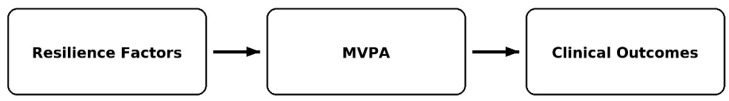
Conceptual pathway of resilience, physical activity, and treatment outcomes.

To address these gaps, this cross-sectional study explored potential resilience factors that could be treatment targets to increase physical activity and treatment outcomes, evaluated associations between resilience constructs, and objectively measured physical activity engagement, physical functioning, and health-related quality of life among adolescents with CMSKP. We propose that resilience factors may be modified to improve MVPA, which would then improve treatment outcomes (see Figure 1). Given the promising qualitative findings and theoretical rationale from our previous work and the broader literature [22,24], this study has two overall aims. First, this study aimed to evaluate potential resilience factors that may be related to physical activity, and relevant treatment outcomes (i.e., functioning and QOL) in order to identify potential treatment targets for future intervention development. Second, we sought to identify whether any resilience variables may be of particular importance for physical activity. We hypothesized that (1) higher resilience factors (e.g., self-efficacy, acceptance, and motivational readiness) would be associated with higher moderate-to-vigorous physical activity (MVPA); (2) higher resilience factors would also be associated with greater quality of life and functioning; and (3) MVPA would be associated with higher quality of life (QOL) and functioning. We also hypothesized that self-efficacy and motivation will be the two most important resilience factors (i.e., associated with the greatest amount of covariability) in MVPA. Several exploratory analyses were also conducted. We explored associations between resilience constructs and other objectively measured physical activity outcomes, including sedentary and light-intensity activities. We also explored associations of other resilience constructs that have not received as much consideration in pediatric pain, including grit, a growth mindset, and affect. This study was designed to generate insights that could inform the development of resilience-focused behavioral interventions aimed at facilitating sustained physical activity engagement, enhancing functional outcomes, and promoting long-term recovery for adolescents living with chronic musculoskeletal pain.

## 2. Methods

### 2.1. Study Design and Overview

This study utilized a cross-sectional observational design to evaluate associations among psychological resilience constructs, objectively measured physical activity, physical functioning, and health-related QOL in adolescents with CMSKP. Adolescents completed validated self-report questionnaires and standardized physical function assessments. Participants also wore accelerometry devices continuously for seven consecutive days. The study and its procedures were conducted in accordance with the Declaration of Helsinki, and approved by the Institutional Review Board of Children’s Mercy Hospital Kansas City (STUDY00001911, approval 6 August 2021). Informed consent was obtained from all subjects involved in the study prior to data collection.

### 2.2. Participants

Participants included 43 adolescents aged 13–18 years diagnosed with CMSKP. CMSKP was defined as persistent pain lasting ≥3 months in muscles, joints, or connective tissues, confirmed by a pediatric pain specialist or rheumatologist. Adolescents were recruited from specialty pediatric rheumatology and chronic pain clinics at a Midwestern pediatric hospital, including adolescents awaiting or recently discharged from intensive interdisciplinary pain treatment (IIPT). Exclusion criteria included chronic pain attributable to medical diseases (e.g., juvenile idiopathic arthritis, cancer), untreated psychiatric disorders (e.g., psychosis, active suicidal ideation), or developmental delays precluding valid assessment completion.

### 2.3. Procedures

Following informed consent and assent, participants completed questionnaires, standardized physical function assessments, and objective physical activity. Participants were asked to wear the activPAL and manually track their sleep for one week following the initial study visit.

### 2.4. Measures

#### 2.4.1. Psychological Resilience Constructs

Domain-Specific Physical Activity Self-Efficacy Questionnaire: This 26-item measure assesses adolescents’ confidence in their ability to engage in physical activity under various circumstances, including during episodes of pain, fatigue, or stress [35]. Items are rated on a 5-point Likert scale (1 = not at all confident, 5 = very confident). Scores are averaged, with higher scores indicating greater physical activity-specific self-efficacy. This measure demonstrates strong psychometric properties in adolescent populations (Cronbach’s α = 0.90–0.95) [35] and α = 0.88–0.98 in this sample.

Acceptance and Fusion Questionnaire for Youth (AFQ-Y): This 17-item measure assesses psychological inflexibility, particularly cognitive fusion (rigid thought patterns) and experiential avoidance (unwillingness to experience discomfort) in pediatric psychological research [36]. Items are rated on a 5-point Likert scale (1 = not at all true, 5 = very true). Higher scores indicate greater psychological inflexibility and lower acceptance, which may translate to lower acceptance of discomfort (e.g., pain, physical activity). The AFQ-Y is a well-validated instrument that has been widely adopted in pediatric psychological research (Cronbach’s α = 0.90) [36] and α = 0.89 in this sample.

Pain Stages of Change Questionnaire—Adolescent version (PSOCQ-A): This 27-item measure assesses adolescents’ motivational readiness to adopt pain self-management strategies, aligning with the Transtheoretical Model stages of Precontemplation, Contemplation, and Action/Maintenance [26]. Items are rated from 1 (strongly disagree) to 5 (strongly agree), with higher scores in Contemplation and Action/Maintenance indicating greater readiness for adopting behavioral changes to manage pain (Cronbach’s α = 0.47–0.9) [37] and α = 0.67–0.83 in this sample.

Positive and Negative Affect Schedule for Children (PANAS-C): The PANAS-C is a 30-item measure assessing the frequency and intensity of positive emotions (e.g., joy, enthusiasm) and negative emotions (e.g., sadness, anxiety) typically experienced by adolescents [38]. Items are rated on a 5-point scale (1 = very slightly/not at all, 5 = extremely) (Cronbach’s α = 0.9). Higher scores indicate greater levels of respective affective dimensions. Internal reliability ranged from α = 0.896–0.910 in this sample.

Revised Life Orientation Test (LOT-R): The LOT-R is a widely used 10-item measure assessing dispositional optimism, defined as generalized positive outcome expectancies [39]. Six scored items and four filler items are rated on a 5-point Likert scale (0 = strongly disagree, 4 = strongly agree), with higher scores reflecting greater optimism (Cronbach’s α = 0.7–0.8) and α = 0.72 in this sample.

Short Grit Scale (Grit-S): The Grit-S is an 8-item validated measure of perseverance and sustained interest toward long-term goals [40]. Items are scored on a 5-point Likert scale (1 = not at all like me, 5 = very much like me), with higher scores reflecting greater perseverance and grit (Cronbach’s α = 0.73–0.83) [40] and α = 0.80 in this sample.

Growth Mindset Scale: This validated 8-item measure (adapted from Dweck, 2006) assesses adolescents’ beliefs about the malleability and development of personal abilities and traits [41]. Items are rated on a 6-point scale (1 = strongly disagree, 6 = strongly agree). Higher scores indicate a stronger growth mindset orientation (Cronbach’s α = 0.83) [42] and α = 0.93 in this sample.

#### 2.4.2. Objective Physical Activity Measurement

ActivPAL3 Monitor: The activPAL3 monitor (PAL Technologies Ltd., Glasgow, UK) is a validated tri-axial accelerometer worn continuously on the anterior thigh for seven consecutive days, capturing detailed patterns of sedentary time, standing, stepping (light and MVPA), steps/day, and sit-to-stand transitions. Participants were instructed to wear the device while sleeping. Data were processed using validated CREA (v1.3) enhanced analysis software algorithms, with minimum criteria for inclusion being ≥4 valid days (≥1 weekend day) and 24 h of daily wear time, allowing 2 h of non-wear time. We classified MVPA as ≥3 METs, which corresponds to ~74 steps/min in the activPAL algorithm (with 120 steps/min ≈ 4 METs); light activity was 1.5–2.99 METs, and sedentary was <1.5 METs [43,44].

#### 2.4.3. Functioning and Quality of Life Measures

Functional Disability Inventory (FDI): This 15-item measure assesses adolescents’ perceived difficulty performing daily activities due to pain [45]. Items are rated from 0 (no trouble) to 4 (impossible), with higher total scores reflecting greater functional disability. The FDI demonstrates strong psychometric properties in pediatric chronic pain populations (Cronbach’s α = 0.86–0.91) [46] and α = 0.88 in this sample. Clinical interpretation of scores is categorized as minimal (0–12), moderate (13–29), and severe disability (≥30), with a 7-point change indicating clinically meaningful improvement or worsening.

Six-Min Walk Test (6MWT): This standardized test, conducted according to American Thoracic Society guidelines, objectively measures aerobic endurance and functional capacity. Adolescents were instructed to walk as far as possible in six minutes along a standardized 30 m course, with total distance recorded in meters [47].

#### 2.4.4. Health-Related Quality of Life and Fatigue Measures

Pediatric Quality of Life Inventory (PedsQL 4.0): The validated 23-item PedsQL assesses overall QOL across physical, emotional, social, and school functioning domains [48]. Items are rated on a 5-point Likert scale (0 = never, 4 = almost always), with higher transformed scores (0–100) indicating better QOL (Cronbach’s α > 0.7) [48] and α = 0.875 in this sample.

PedsQL Multidimensional Fatigue Scale: This validated 18-item measure assesses fatigue-related impairment across three domains: general fatigue, sleep/rest fatigue, and cognitive fatigue [49]. Items are rated on the same 5-point scale as the core PedsQL. Higher scores indicate less fatigue-related impairment (Cronbach’s α = 0.8–0.9) [50] and α = 0.64–0.89 in this sample. The lowest reliability (0.642) was found for cognitive fatigue; the other subscales were above α = 0.88.

### 2.5. Statistical Analyses

Analyses were conducted using SPSS (version 28.0, IBM Corp., Armonk, NY, USA). Descriptive statistics summarized demographics and all study measures. Spearman’s rho correlation coefficients assessed relationships between resilience constructs (self-efficacy, acceptance, motivation), physical activity outcomes (sedentary behavior, MVPA, light activity), physical functioning measures (FDI, 6MWT), and QOL (PedsQL total scores). Statistical significance was defined at *p* < 0.05 (two-tailed). Bias-corrected 95% bootstrap confidence intervals (CIs) were estimated (5000 resamples) to provide robust CI estimates given the modest sample size. A hierarchical linear regression, with stepwise forward entry of the five resilience factors with the highest correlations with MPVA; based on previous literature, we also included motivation (pre-contemplation). Additional exploratory analyses examined correlations between supplementary psychosocial constructs (optimism, grit, growth mindset) and primary study outcomes to contextualize resilience and physical activity relationships.

## 3. Results

### 3.1. Participant Characteristics

Forty-three adolescents with clinician-confirmed CMSKP participated in this study (mean age = 16.22 years, *SD* = 1.33, range 14.06–18.82). The sample was predominantly assigned female at birth (88.4%) and primarily identified as White (83.7%), with additional racial identities including multiracial (14.0%) and American Indian/Alaska Native (2.3%). Approximately 18.6% identified as Hispanic or Latino. Regarding gender identity, 62.8% identified as female, 11.6% as male, and 25.6% identified with a non-cisgender identity, including transgender, nonbinary, genderqueer, agender, or another identity. See Table 1 for demographic details.

### 3.2. Descriptive Statistics of Primary Variables

Participants reported moderate levels of functional disability (FDI; *M* = 16.53, *SD* = 9.72). Adolescents spent most of their waking hours sedentary (*M* = 630.09 min/day, *SD* = 86.99). They engaged in limited light physical activity (*M* = 215.90 min/day, *SD* = 81.12) and MVPA (*M* = 11.68 min/day, *SD* = 9.36). Aerobic capacity measured by the six-minute walk test (6MWT) yielded a mean walking distance of 463.73 m (*SD* = 90.48), reflecting below-average endurance compared to normative values for healthy adolescents, who typically walk between 540 and 620 m [13,32]. Descriptive statistics for physical activity, functioning, and resilience measures are summarized in Table 2.

Participants reported moderate pain levels (*M* = 4.68, *SD* = 1.87 on a 0–10 scale) over the previous two weeks. Pain-related self-efficacy varied by domain, with higher confidence for ambulatory tasks at school (*M* = 8.78, *SD* = 2.48) and lower confidence for transportation and school attendance (*M* = 5.81, *SD* = 2.91). Acceptance, as assessed by the Avoidance and Fusion Questionnaire for Youth (AFQ-Y), indicated moderate psychological flexibility (*M* = 18.70, *SD* = 11.35). Participants most frequently aligned with the contemplation stage of motivation (*M* = 35.47, *SD* = 5.32) as assessed by the Pain Stages of Change Questionnaire—Adolescent version (PSOCQ-A), reflecting a willingness to consider and engage in self-management strategies for pain.

### 3.3. Associations Between Psychological Resilience Factors and Physical Activity

Correlational analyses revealed several notable associations between adolescents’ physical activity engagement and resilience-related psychological constructs (see Table 3). Greater self-efficacy for physical activity across several domains of functioning, including school, household activities, leisure time, school ambulation, and transportation were associated with higher levels of MVPA (range: *ρ* = 0.443–0.599, *p* < 0.05). However, light physical activity was only related to physical activity; self-efficacy for household activity (*ρ* = 0.345, *p* = 0.031) and leisure time (*ρ* = 0.354, *p* = 0.027) were significantly associated with higher light physical activity; physical activity self-efficacy at school (*ρ* = 0.303, *p* = 0.061) and school ambulation (*ρ* = 0.299, *p* = 0.064) were only marginally associated with light physical activity. The pre-contemplation stage of motivation was significantly associated with less sedentary time (*ρ* = −0.389, *p* = 0.014) but did not approach significance with any other resilience or functional measure.

### 3.4. Associations Between Psychological Resilience Factors and Functioning Outcomes

Additional notable associations between psychological resilience constructs and measures of aerobic capacity, physical functioning, and health-related QOL were identified (Table 4). Several domains of self-efficacy, including school activity (*ρ* = 0.388, *p* = 0.010), household activity (*ρ* = 0.434, *p* = 0.004), leisure time (*ρ* = 0.463, *p* = 0.002), ambulation at school (*ρ* = 0.573, *p* < 0.001), and ambulatory transport (*ρ* = 0.485, *p* < 0.001) were associated with higher functional capacity (i.e., the 6MWT). Self-efficacy (school, household, leisure time, ambulation at school, ambulatory transport, *p* < 0.01), general fatigue (*ρ* = −0.427, *p* = 0.004), sleep and rest fatigue (*ρ* = −0.378, *p* < 0.01), the pre-contemplation stage of motivation (*ρ* = 0.420, *p* = 0.005), and the maintenance stage of motivation (*ρ* = −0.489, *p* = < 0.001) were significantly associated with daily physical functioning (i.e., the Functional Disability Inventory). However, other measures of resilience (i.e., affect, grit, optimism, or growth mindset) were not related to FDI. Resilience factors including motivation/pre-contemplation (*ρ* = −0.454, *p* = 0.002), maintenance of change (*ρ* = 0.327, *p* = 0.033), optimism (*ρ* = 0.535, *p* < 0.001), positive affect (*ρ* = 0.482, *p* = 0.001), flexibility/greater acceptance (*ρ* = −0.503, *p* < 0.001), and negative affect (*ρ* = −0.529, *p* < 0.001) were also associated with QOL.

### 3.5. Associations Among Physical Activity, Function, and Quality of Life

Bivariate analyses revealed several significant associations among physical activity metrics, physical function, and health-related QOL (see Table 4). Greater aerobic capacity (i.e., 6MWT distance; *ρ* = 0.48, *p* = 0.002) and functional disability (i.e., FDI; *ρ* = −0.56, *p* < 0.001) were significantly associated with MVPA, while the Pediatric Quality of Life Inventory (PedsQL) was not related to MVPA (*ρ* = 0.20, *p* = 0.22). The 6MWT distance, FDI scores, and PedsQL scores were not related to light-intensity activity or sedentary behavior, underscoring that MVPA uniquely relates to improved functional outcomes and lower disability levels among adolescents with CMSKP.

### 3.6. Linear Regression Predicting MVPA

A two-step hierarchical, stepwise linear regression was conducted with age and biological sex in step 1, and with resilience factors entered into the second step, with stepwise entry. Factors included self-efficacy for physical activity (e.g., PA at school, home, during leisure time, ambulation at school, self-transportation), and motivation (i.e., pre-contemplation). However, only self-efficacy for ambulation at school was retained in the regression model (*B* = 0.42, *p* = 0.008) (see Table 5).

## 4. Discussion

This study expands upon prior qualitative work (In Press) [34] and conceptual research by quantitatively examining the relationships between several measures of psychological resilience, objectively measured physical activity, functional outcomes, and health-related QOL among adolescents with CMSKP. By exploring and identifying resilience factors that may be related to an established, modifiable treatment target—such as MPVA—and may be related to better outcomes across multiple domains of functioning, this study aimed to identify specific resilience factors that could be evaluated in future research and intervention development. Our main hypotheses were partially confirmed: self-efficacy and functional outcome measures were significantly associated with greater MVPA, but generally less so with light and sedentary activity. However, relationships with other physical activity intensity levels were less clear or were related to only some variables. Surprisingly, motivation was generally unrelated to physical activity, with the exception of pre-contemplation for change and sedentary time.

Among resilience constructs evaluated, physical activity self-efficacy emerged as the strongest correlate of both physical activity engagement and functional outcomes. Adolescents reporting higher self-efficacy demonstrated significantly greater MVPA engagement, better aerobic endurance, and lower disability levels. This finding strongly supports existing literature, identifying self-efficacy as one of the most consistent psychological factors associated with sustained behavioral engagement and improved functioning in pediatric chronic pain populations [24,27,35,51]. Adolescents reporting greater confidence in their ability to engage in physical activity despite pain demonstrated objectively greater physical activity and superior functional outcomes, suggesting self-efficacy as a potential modifiable target in resilience-focused interventions aimed at adolescents with chronic pain. Interestingly, when considering results from the stepwise hierarchical regression, only self-efficacy for ambulation at school was retained in the model. This suggests that interventions to improve how adolescents navigate their school environment may have the greatest initial utility to help improve their overall MVPA.

This study also sought to identify resilience factors that may be related to better functioning, as they may also be good targets for future intervention development. Adolescents who actively accepted their pain experience while pursuing valued activities (per the AFQ-Y) reported higher levels of well-being. This is consistent with prior findings identifying acceptance-based coping as critical in facilitating sustained engagement in meaningful activities even with ongoing pain [36,52]. Acceptance-based therapeutic approaches may enhance adolescents’ capacity to maintain functional engagement and improve overall psychosocial outcomes [21,25,36,53], including physical activity goals [54].

Motivational readiness, assessed using the Pain Stages of Change Questionnaire—Adolescent version (PSOCQ-A), provides additional insights into adolescents’ readiness to adopt pain self-management behaviors [22,26,55]. Motivation was generally unrelated to physical activity, but did track with one outcome variable—QOL. Higher pre-contemplation scores, reflecting adolescents lack of consideration or preparation for behavior change, were significantly associated with poorer perceived QOL. Conversely, those high in reported maintenance (i.e., working to sustain behavioral change) were linked to higher perceived QOL. This result may well reflect our recruitment strategy, targeting adolescents from active specialty clinical environments, including those recently discharged or awaiting intensive interdisciplinary pain treatment (IIPT). Adolescents recruited from these contexts may have elevated motivational readiness compared to adolescents without similar clinical experiences, suggesting motivational readiness could represent a critical marker for intervention timing, tailoring, and personalization to optimize clinical effectiveness.

Interestingly, when considering the range of physical activity intensities—sedentary behavior, light-intensity, and MVPA—there were some differences in which resilience measures were related different intensity levels of PA. While MVPA was significantly and most strongly related to self-efficacy, light physical activity was also related to self-efficacy and grit, and it approached significance with both optimism and having a growth mindset; conversely, sedentary time was related to the pre-contemplation stage of motivation. While these analyses are underpowered and exploratory, these differences across physical activity intensities may capture different aspects and stages of individuals in their pain journey and could yield useful treatment-related information to aid adolescents across their journey. It may be that those who are earlier in their journey may be mustering momentum to begin changing (precontemplation); in the meantime, they are sedentary, with poorer QOL and heightened functional disability. Comparatively, those who are midway through their journey (and perhaps beginning by increasing light physical activity) are working to obtain treatment gains, and resilience factors like grit and optimism may be more relevant mid-treatment. Those who are further in their journey are more established in self-management and have likely developed self-efficacy for physical activity over time. In line with the common treatment adage that “function improves before symptoms improve,” with physical activity as one of the main drivers of this mechanism, those further in their journey likely reap the benefits during the maintenance stage, which present as improved QOL and reduced functional disability. These potential relationships need to be evaluated further in a larger, more rigorous study, over a longitudinal timeframe to infer causal mechanisms. Although previous literature has identified reducing sedentary time as clinically beneficial—given associations with mood, fatigue, and physical deconditioning [56]—our findings support continued focused on MVPA engagement as a primary target. However, further work is needed to evaluate the impacts of different physical activity intensities, as reducing sedentary behaviors and increasing light activity may be helpful in developing treatment momentum and fostering self-efficacy, which could contribute to increase MPVA engagement. It is possible that in this study, given that we recruit from those seeking treatment in pain clinics, our sample may already be attempting to engage in greater MVPA, as they have established and are engaged in active care. Future work should consider treatment related variables including time since diagnosis, length of time engaged in current treatment, and previously received treatment, which could all impact their activity levels.

## 5. Clinical Implications

Our findings may inform future work in clinical care in several ways. Primarily, results lend support to the value of incorporating resilience-enhancing strategies for pediatric chronic pain management programs to enhance adolescents’ adherence to MVPA—potentially through increased self-efficacy. Further, interventions explicitly aimed at strengthening self-efficacy, fostering pain acceptance, and leveraging adolescents’ motivational readiness for behavior change may be considered as strategies to enhance treatment outcomes across the continuum of care—particularly to improve QOL. Self-efficacy-driven strategies could include graded exposure strategies, guided mastery experiences for engaging in different physical activity activities, goal-setting interventions, self-monitoring strategies, and structured feedback to enhance adolescents’ confidence in their physical capabilities. Providers often utilize a mixture these strategies throughout treatment but may not do so as part of a set of strategies for the purpose of increasing self-efficacy. There may be utility in explicitly drawing attention to these strategies are part of standard care. Other strategies, such as learning to engage in optimistic thinking and building grit, while not clearly related to outcomes in this study, may be used to support the development of self-efficacy; other potential resilience augments should be explored. The influence of these strategies should be evaluated in future studies designed to examine their effect over a longitudinal time-course, pre-treatment, during treatment, and then post treatment.

Relatedly, our results also provide some support for the use of existing pain strategies, as long as they aim toward physical activity engagement specifically. Given the significant relationship between pain acceptance and QOL outcomes, acceptance-based interventions—such as Acceptance and Commitment Therapy (ACT)—may enhance adolescents’ capacity to engage in meaningful physical activities despite pain, thereby reducing disability and enhancing overall psychosocial well-being. Exploration of values may help increase engagement in these meaningful activities, and tailoring physical activities to individual preference and values can help to increase sustainability and adherence. Finally, our findings suggest that motivational readiness should be considered to personalize intervention approaches particularly when thinking about initiating and developing momentum for physical activity. While higher levels of MVPA may be the eventual treatment target, there may be utility for some participants in targeting less intense physical activity engagement to initiate behavior change and begin to develop self-efficacy, whether as a strategy to work with low motivation, fear of injury, or self-efficacy.

## 6. Limitations and Future Directions

While this study has several strengths in its measurement of objective physical activity, inclusion of a range of resilience factors, and inclusion of multiple, clinically relevant patient-reported outcome measures (i.e., FDI, PEDS-QL), there are several limitations that must be acknowledged. First, our cross-sectional design precludes establishing the direction of relationships among resilience constructs, physical activity engagement, and clinical outcomes. For example, the observed link between self-efficacy and MVPA engagement is correlational and may be representative of reciprocal determinism [57,58], rather than self-efficacy simply driving MVPA engagement. While fostering self-efficacy in physical activity is likely to promote sustained behavioral change (in this case, increased MVPA), self-efficacy is also impacted by past performance. It may be that improvements in MVPA in turn, could lead to greater mastery of physical activity, and therefore be likely to promote self-efficacy. Although identified associations align well with existing theoretical frameworks highlighting the role of resilience in pain management [18,21], longitudinal and intervention-based studies are needed to clarify causal pathways and directionality. Treatment-related studies may provide evidence of causal resilience mechanisms and demonstrate whether resilience training impacts functioning and treatment mechanisms (e.g., MVPA). Second, while we assess MVPA, we do not differentiate between different types of activity (e.g., planned exercise vs. higher-intensity incidental exercise). Given our goal of identifying resilience factors specific to physical activity engagement, there could be different resilience factors that may be related to physical activity versus planned exercise. Additionally, there may be confounding variables that could affect relationships, which are not considered with the bi-variate correlations in this study (e.g., age, gender, pain intensity); potential confounds should be evaluated more completely in a multivariate study with a larger sample size.

Third, our sample was recruited predominantly from specialty clinical settings, which may limit the generalizability of findings to broader adolescent populations with chronic pain. While our predominantly female sample is representative of pain clinics, our results may underrepresent or miss resilience characteristics that could be unique to boys. Our sample may also represent a higher motivated subset, given that they are engaged in pain care and were willing to volunteer for a research study. Future research should incorporate diverse samples, including community-based adolescents or those at earlier stages in their clinical journeys, to enhance external validity. Fourth, this study was developed and conducted as an initial pilot to help identify potentially relevant resilience factors; it is underpowered to draw definitive conclusions and in need of repetition with a larger sample size and was also prohibitive of more complex and suitable methods (e.g., regression analyses, mediation analyses). Lastly, while several resilience constructs were evaluated, future research should explore additional psychosocial variables (e.g., emotion regulation, social support, stress exposure, kinesiophobia, catastrophizing) to fully elucidate resilience and psychosocial mechanisms influencing physical activity and functional outcomes in adolescents with chronic pain.

## 7. Conclusions

The findings of this study align with the existing literature highlighting the unique importance of MVPA in pain severity, physical functioning, and psychosocial outcomes among pediatric chronic pain populations [16,17,59], and provides support for psychological resilience constructs—particularly self-efficacy—as potential facilitators of MVPA. Other resilience factors—such as acceptance and motivational readiness—may also be useful treatment targets to improve functioning and QOL in adolescents with chronic musculoskeletal pain. Incorporating targeted, resilience-enhancing interventions within multidisciplinary pediatric pain management programs could improve adolescents’ treatment adherence, functional outcomes, and overall psychosocial well-being. Ultimately, these findings point to the potential value of developing strengths-based interventions that support adolescents’ confidence in physical activity, promote acceptance-based coping, and leverage motivational readiness to encourage sustained physical activity and promote recovery.

## Figures and Tables

**Table 1:** Participant demographics.

Variable	Value (*n* = 43)
Age (mean ± SD)	*M* = 16.22 ± 1.33 Range = 14.06–18.82
Biological Sex at Birth	
Female	88.4% (*n* = 38)
Male	11.6% (*n* = 5)
Race	
White	83.7% (*n* = 36)
More than one race	14.0% (*n* = 6)
American Indian/Alaska Native	2.3% (*n* = 1)
Ethnicity	
Hispanic or Latino	18.6% (*n* = 8)
Non-Hispanic	81.4% (*n* = 35)
Gender Identity	
Female	62.8% (*n* = 27)
Male	11.6% (*n* = 5)
Trans male/Trans man	9.3% (*n* = 4)
Genderqueer/Gender nonconforming	4.7% (*n* = 2)
Nonbinary	4.7% (*n* = 2)
Agender	2.3% (*n* = 1)
Other/different identity	4.7% (*n* = 2)

**Table 2:** Descriptive statistics for measures.

Domain	Variable	N	Mean	SD	SE	95% CI Lower	95% CI Upper
Physical Activity and Functioning							
	Total Time Sedentary (min)	39	630.09	86.99	13.93	602.97	565.73
	Total Time Light PA (min)	39	215.90	81.12	12.99	193.41	241.67
	Total Time MVPA (min)	39	11.68	9.36	1.50	8.99	14.79
	6 min Walk (m)	43	463.73	90.48	13.80	437.31	490.24
	Functional Disability Inventory Total	43	16.53	9.72	1.48	13.79	19.37
	PedsQL—Total Quality of Life	43	46.80	16.32	2.49	41.99	51.49
	PedsQL—General Fatigue	43	45.89	21.27	3.24	39.98	51.86
	PedsQL—Sleep and Rest Fatigue	43	47.00	17.14	2.61	41.48	52.13
	PedsQL—Cognitive Fatigue PedsQL	43	47.09	21.18	3.23	40.99	53.00
Physical Activity Self-Efficacy							
	PA Self-Efficacy School	43	5.81	2.97	0.45	4.92	6.72
	PA Self-Efficacy Transportation	43	5.81	2.91	0.44	4.91	6.66
	PA Self-Efficacy Household	43	7.16	1.93	0.29	6.57	7.71
	PA Self-Efficacy Leisure Time	43	6.74	2.75	0.42	5.91	7.51
	PA Self-Efficacy Ambulatory at School	43	8.78	2.48	0.38	7.98	9.50
	PA Self-Efficacy Ambulatory Transport	43	8.61	2.57	0.39	7.84	9.32
Motivation							
	Pre-Contemplation	43	19.07	6.12	0.93	17.30	20.88
	Contemplation	43	35.47	5.32	0.81	33.91	36.98
	Action	43	23.30	3.23	0.49	22.33	24.23
	Maintenance	43	28.14	4.03	0.61	27.05	29.23
Other Resilience Measures							
	Acceptance Fusion Questionnaire Total	43	18.70	11.35	1.73	15.41	22.16
	Revised Life Orientation Test–Optimism	43	12.37	3.58	0.55	11.33	13.42
	PANAS–Positive Affect	43	39.60	8.67	1.32	37.02	42.35
	PANAS–Negative Affect	43	35.19	11.01	1.68	32.07	38.40
	Total Grit	43	3.05	0.57	0.09	2.88	3.22
	Growth Mindset	43	4.21	1.40	0.21	3.82	4.57

**Table 3:** Correlations between physical activity, functioning, and resilience factors.

	Sed. Time			Light PA			MVPA		
	rho	95% CI		rho	95% CI		rho	95% CI	
6 Min Walk	0.051	−0.278	0.369	0.088	−0.243	0.401	0.480 **	0.184	0.696
FDI	−0.058	−0.376	0.271	−0.153	−0.454	0.180	−0.557 **	−0.746	−0.284
PedsQL	0.225	−0.107	0.512	0.227	−0.105	0.514	0.199	−0.134	0.491
PedsQL—General Fatigue	0.192	−0.141	0.486	0.215	−0.117	0.504	0.284	−0.044	0.557
PedsQL—Sleep and Rest Fatigue	0.257	−0.073	0.537	0.015	−0.311	0.337	0.179	−0.154	0.476
PedsQL—Cognitive Fatigue	0.115	−0.217	0.423	0.289	−0.039	0.561	0.044	−0.284	0.363
PA Self-Efficacy School	0.133	−0.200	0.438	0.303	−0.023	0.571	0.504 **	0.215	0.712
PA Self-Efficacy Transportation	0.214	−0.119	0.503	0.083	−0.248	0.396	0.307	−0.019	0.574
PA Self-Efficacy Household	0.030	−0.297	0.351	0.345 *	0.023	0.602	0.468 **	0.169	0.688
PA Self-Efficacy Leisure Time	−0.026	−0.347	0.301	0.354 *	0.034	0.608	0.460 **	0.160	0.683
PA Self-Efficacy Ambulatory at School	−0.009	−0.332	0.316	0.299	−0.028	0.568	0.599 **	0.341	0.773
PA Self-Efficacy Ambulatory Transport	0.067	−0.263	0.383	0.253	−0.078	0.533	0.443 **	0.139	0.671
PSOCQ—Pre-Contemplation	−0.389 *	−0.633	−0.074	0.147	−0.186	0.449	−0.070	−0.386	0.260
PSOCQ—Contemplation	−0.162	−0.462	0.171	0.094	−0.238	0.405	0.050	−0.279	0.368
PSOCQ—Action	0.111	−0.221	0.420	0.072	−0.258	0.387	−0.031	−0.352	0.296
PSOCQ—Maintenance	0.273	−0.057	0.548	0.141	−0.192	0.445	0.091	−0.240	0.403
Acceptance Fusion Questionnaire Total	−0.226	−0.513	0.106	−0.002	−0.326	0.323	0.037	−0.291	0.357
Revised Life Orientation Test—Optimism	0.142	−0.191	0.446	0.072	−0.259	0.387	−0.020	−0.342	0.306
PANAS—Positive Affect	−0.149	−0.452	0.184	0.251	−0.079	0.532	0.006	−0.319	0.330
PANAS—Negative Affect	−0.121	−0.429	0.211	−0.117	−0.425	0.215	0.038	−0.290	0.358
Total Grit	0.137	−0.196	0.442	−0.385 *	−0.631	−0.070	−0.080	−0.394	0.251
Growth Mindset	0.189	−0.144	0.484	−0.291	−0.562	0.036	−0.068	−0.384	0.262

* *p* < 0.05; ** *p* < 0.01.

**Table 4:** Correlations between functioning and resilience measures.

	6 Min Walk			FDI			PEDS-QL		
	rho	95% CI		rho	95% CI		rho	95% CI	
PA Self-Efficacy School	0.388 *	0.090	0.622	−0.419 **	−0.645	−0.127	0.224	−0.091	0.498
PA Self-Efficacy Transportation	0.269	−0.043	0.533	−0.281	−0.543	0.030	0.180	−0.137	0.463
PA Self-Efficacy Household	0.434 **	0.144	0.655	−0.565 **	−0.744	−0.311	0.223	−0.092	0.497
PA Self-Efficacy Leisure Time	0.463 **	0.181	0.675	−0.460 **	−0.673	−0.177	0.145	−0.171	0.435
PA Self-Efficacy Ambulatory at School	0.573 **	0.321	0.749	−0.530 **	−0.721	−0.264	0.175	−0.142	0.459
PA Self-Efficacy Ambulatory Transport	0.485 **	0.207	0.690	−0.446 **	−0.664	−0.160	0.241	−0.073	0.512
PSOCQ—Pre-Contemplation	−0.284	−0.545	0.027	0.420 **	0.128	0.645	−0.454 **	−0.669	−0.169
PSOCQ—Contemplation	0.004	−0.305	0.312	0.137	−0.179	0.428	−0.037	−0.342	0.275
PSOCQ—Action	0.188	−0.128	0.469	−0.149	−0.437	0.168	0.225	−0.090	0.499
PSOCQ—Maintenance	0.253	−0.061	0.521	−0.489 **	−0.693	−0.213	0.327 *	0.020	0.577
Acceptance Fusion Questionnaire Total	−0.109	−0.404	0.207	0.273	−0.039	0.537	−0.503 **	−0.703	−0.231
Revised Life Orientation Test—Optimism	−0.054	−0.357	0.259	−0.145	−0.434	0.171	0.535 **	0.271	0.724
PANAS—Positive Affect	0.031	−0.281	0.336	−0.134	−0.425	0.182	0.482 **	0.203	0.688
PANAS—Negative Affect	0.083	−0.232	0.382	0.129	−0.187	0.421	−0.529 **	−0.720	−0.263
Total Grit	−0.203	−0.481	0.113	0.110	−0.206	0.405	−0.154	−0.442	0.162
Growth Mindset	−0.030	−0.336	0.281	0.013	−0.297	0.321	0.168	−0.148	0.453

* *p* < 0.05; ** *p* < 0.01.

**Table 5:** Linear regression predicting moderate-to-vigorous physical activity.

	Predictor	B	SE	β	t	*p*	95% CI Lower	95% CI Upper
Step 1								
	Intercept	−18.78	17.6	—	−1.07	0.29	−54.47	16.91
	Age	1.9	1.08	0.28	1.75	0.09	−0.3	4.09
	Sex	−2.19	4.41	−0.08	−0.5	0.62	−11.12	6.75
								
Step 2								
	Intercept	−23.239	16.205	—	−1.434	0.160	−56.136	9.659
	Age	1.351	1.010	0.199	1.337	0.190	−0.700	3.403
	Sex	−3.410	4.062	−0.123	−0.840	0.407	−11.657	4.836
	PA Self-Efficacy, Ambulatory at School	1.556	0.555	0.420	2.806	0.008	0.430	2.683

## Data Availability

Data will be made available per NIH policy at the conclusion of the study.
